# Palladium supported on polypyrrole/reduced graphene oxide nanoparticles for simultaneous biosensing application of ascorbic acid, dopamine, and uric acid

**DOI:** 10.1038/s41598-020-59935-y

**Published:** 2020-02-19

**Authors:** Buse Demirkan, Sait Bozkurt, Kemal Cellat, Kubilay Arıkan, Mustafa Yılmaz, Aysun Şavk, Mehmet Harbi Çalımlı, Mehmet Salih Nas, Mehmet Nuri Atalar, Mehmet Hakkı Alma, Fatih Sen

**Affiliations:** 1 Sen Research Group, Department of Biochemistry, Faculty of Arts and Science, Dumlupinar University, Evliya Çelebi Campus, 43100 Kütahya, Turkey; 2 Department of Environmental Engineering, Faculty of Engineering, University of Igdir, Igdir, Turkey

**Keywords:** Biocatalysis, Electrocatalysis, Nanoparticles, Electronic properties and devices

## Abstract

In this study, we report a facile and effective production process of palladium nanoparticles supported on polypyrrole/reduced graphene oxide (rGO/Pd@PPy NPs). A novel electrochemical sensor was fabricated by incorporation of the prepared NPs onto glassy carbon electrode (GCE) for the simultaneous detection of ascorbic acid (AA), dopamine (DA) and uric acid (UA). The electrodes modified with rGO/Pd@PPy NPs were well decorated on the GCE and exhibited superior catalytic activity and conductivity for the detection of these molecules with higher current and oxidation peak intensities. Simultaneous detection of these molecules was achieved due to the high selectivity and sensitivity of rGO/Pd@PPy NPs. For each biomolecule, well-separated voltammetric peaks were obtained at the modified electrode in cyclic voltammetry (CV) and differential pulse voltammetry (DPV) measurements. Additionally, the detection of these molecules was performed in blood serum samples with satisfying results. The detection limits and calibration curves for AA, DA, and UA were found to be 4.9 × 10^−8^, 5.6 × 10^−8^, 4.7 × 10^−8^ M (S/N = 3) and ranging from 1 × 10^−3^ to 1.5 × 10^−2^ M (in 0.1 M PBS, pH 3.0), respectively. Hereby, the fabricated rGO/Pd@PPy NPs can be used with high reproducibility, selectivity, and catalytic activity for the development of electrochemical applications for the simultaneous detection of these biomolecules.

## Introduction

Human body fluids contain very important biomolecules such as ascorbic acid (AA), dopamine (DA) and uric acid (UA), which are very effective in life functions. AA, also called vitamin C, effects as an antioxidant, present in human nutrition, and can be soluble in water. AA plays an important role in biological activities as a reducing agent for the protection of the human body against to side effects of the oxidation process. AA also involved in the activities of living structures such as blood vessels, cell development, bone-cartilage, collagen synthesis, and healing of burns and wounds. Additionally, AA is effective for the prevention and treatment of various diseases, including cancer, mental illness, AIDS, infertility, and colds^1–5^. DA, as an organic cation, is found in the brain and body fluids of tissues. DA acts as a neurotransmitter in the functions of cardiovascular, hormonal, and nervous systems; it has a vital regulating function in cognitive systems. Various neurological disorders and diseases such as Schizophrenia, Parkinson, and Huntington are taken place due to the lack of DA^6–8^. UA is produced as a result of purine metabolism, detection of UA in the products of biological functions indicates the presence of various diseases. For this reason, determining the levels of UA in body fluids is used in the diagnosis of various diseases and disorders. These diseases include gout, kidney disease, Lesch-Nyhan syndrome, and organic acidemia. It is known that high uric acid levels in biological fluids can cause kidney diseases, hyperuricemia, hypertension, and cardiovascular diseases, low levels of UA can cause Wilson’s disease, Fanconi syndrome, celiac disease^9,10^.

Due to the importance of these molecules, detection methods need to be improved for the faster, repeatable, and simultaneous detection^11^. The presence of AA in a highly oxidized concentration with an almost equal amount to DA on bare electrodes is a huge problem in detection studies. The main reason for this issue is that AA reacts with the products of oxidation DA that leads to enhance the amount of DA. This makes challenging the detection of DA and AA simultaneously^12–14^.

In biological fluids, DA, UA, and AA co-exist and, due to the similarity of their oxidation potentials, it is difficult to conduct a simultaneous determination. Various analytical methods such as spectrophotometry^15^, electrochemical^16^, enzymatic-colorimetric^17,18^, fluorescence^16,19^, high-performance liquid chromatography (HPLC)^20^, and chemiluminescence^16^ were used to determine these three bioactive molecules together. However, the applicability of these techniques relatively difficult and requires high cost in most cases; for these reasons, it is required to develop new methods to achieve easy utilization, cost-effective, and reusable. Due to the similarity of the chemical composition and structures of AA, DA, and UA, on bare electrodes, their electrochemical responses are overlapped. To overcome this challenge, our research group focused on the development of novel electrodes that modified with rGO/Pd@PPy NPs for improved detection limits and simultaneous electrochemical determination of AA, DA, and UA.

Recently, the use of GCE in electrochemistry and surface modification are becoming important. Different metallic nanostructured materials such as Au, Ni, Cu have been comprehensively used to develop electrochemical sensors considering their good electrochemical and eco-friendly properties. However, using these mono-metallic materials might have some disadvantages such as low selectivity on detecting biomolecules, relatively low catalytic activity, and high cost^21^. In order to enhance catalyst properties of mono-metallic components, supporting them onto a carbonaceous material provides a more porous structure for the enhanced surface area, lowered the charge transfer resistance along with the increased electrocatalytic performance and lower the total cost. Herein, reduced graphene oxide (rGO) has attracted great attention due to its unique qualities such as mechanical and chemical stability, thermal properties, and excellent conductivity. rGO contains certain amounts of defects and functional groups that have oxygen. These oxygen-containing functional groups enable adsorption and pre-concentration of the redox species (AA, UA, DA in this case) and effectively catalyze the redox reactions. rGO-assisted hybrid nanocomposites have been extensively studied in chemical sensor technology, potential applications in energy storing and catalysts. In these studies, Pd^22^, Pt^23^, Ru^24^, Rh^25^, Zn^26^, Co^11^, and Au^27^ are used as a conductive matrix of nanocomposite materials. The composite structures created in this way have proven to have excellent electrical conductivity, high chemical stability, larger surface area, high mechanical strength^28^. Among these metallic nanoparticles, Pd takes attention due to its excellent catalytic capabilities^29^. Polypyrrole (PPy), a conductive polymer, can be combined with the nanoparticles and it has superior properties such as biocompability, easy synthesis, high electrochemical and thermal stability, and good electronic characteristics. Moreover, the amine group on the pyrrole ring may lead to enhancement of biomolecular sensing. Therefore, a sensor design modified using Palladium nanoparticles, rGO, and PPy which is one of the most extensively used conductive polymer, can bring together the advantages of each component and provide a promising electrochemical sensor.

**Figure 1:**
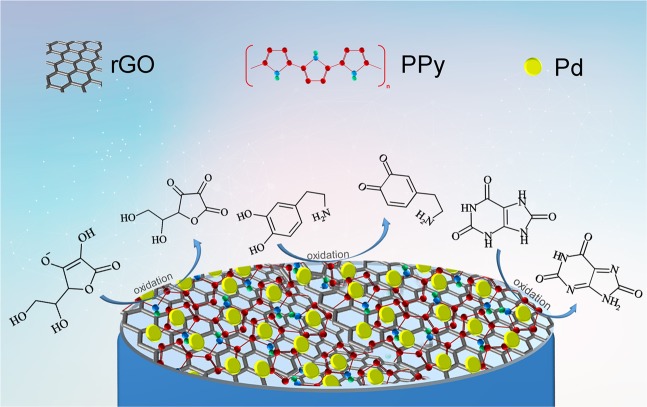
The schematic diagram for the electrochemical oxidation process of AA, DA and UA at the surface of rGO/Pd@PPy biosensor.

In our study, rGO/Pd@PPy NPs prepared for linking the benefits of rGO, Pd and PPy, and they were plated onto a GCE for simultaneous detection of AA, DA, and UA (Fig. 1). TEM, SEM, XRD, Raman, and XPS analysis were used to the characterization of rGO/Pd@PPy NPs. Herein, the electrochemical characteristics of rGO/Pd@PPy/GCE, for the individual and simultaneous detection of AA, DA, and UA, were investigated by cyclic voltammetry (CV) and differential pulse voltammetry (DPV) techniques. The electrodes modified with rGO/Pd@PPy NPs were utilized for the simultaneous detection of AA, DA, and UA exhibited high selectivity in serum samples.

## Experimental

### Chemicals

All the chemicals and solutions were analytical grade and used without a further purification process. AA, DA, UA, Nafion, graphite powder, Polypyrrole (PPy), Palladium chloride (PdCl_2_), Ethyl alcohol (99% pure alcohol), and N, N D-dimethylformamide (DMF, 99.8% anhydrous) were obtained from Sigma Aldrich. Ammonium persulfate (APS) was obtained from Merck. Water used in the experiments purified with a Millipore water purification system (18 MΩ). Phosphate buffered saline (PBS, 0.1 M) was adjusted with HCl acid at different pHs.

### Preparation of rGO/Pd@PPy NPs

Graphene oxide (GO) was synthesized according to the Hummer’s method^30,31^, which is given in supporting information in detail. GO (40 mg) and cetrimonium bromide (91 mg) were added to an aqueous dispersion of GO nanocomposite before polymerization under ultrasonication. Phosphoric acid (15 ml) and the required amount of pyrrole monomer were added gradually and stirred about 2 hours by keeping the temperature 15 °C. For the polymerization, an aqueous solution of ammonium persulfate (APS) was slowly added to the mixture. The polymerization was carried out at for 4 hours at a controlled temperature of 15 °C. The resulting black precipitates were filtered, washed with methanol, and dried under vacuum. In order to prepare rGO, the dried sample of GO was mixed with hydrazine hydrate. rGO/Pd@PPy (1:1) nanocomposites were obtained by the following procedure: 20 mg rGO/PPy powder was dispersed in ethylene glycol (30 mL) for 1 hour by ultrasonic treatment, and 7 mg/mL PdCl_2_ solution was added with the help of vigorously stirring. The pH of this solution was adjusted to 7 by the addition of 1 M NaOH. Then, a microwave-assisted procedure was conducted using a conventional microwave oven (1000 W, 2.45 GHz) at 30-second intervals for 2 minutes. The resulting slurry was centrifuged, washed with deionized water and allowed to dry at 100 °C under vacuum.

### Preparation of the modified electrode with rGO/Pd@PPy NPs

Firstly, the working electrode was cleaned with alumina, washed with ethanol, and deionized water. The obtained rGO/Pd@PPy NPs (8 µL) was dropped on the working electrode, as explained in the supporting information section. Thus, the working electrode modified with rGO/Pd@PPy was prepared for CV and DPV measurements.

### Experimental setup and characterization of nanoparticles

Transmission electron microscopy (TEM, JEOL 200 kV) analyses were conducted to investigate the surface morphology of rGO/Pd@PPy and palladium distribution on support material (PPy/rGO). Prior to TEM analysis, rGO/Pd@PPy NPs were dispersed into ethanol with sonication for a few minutes, poured onto a carbon-coated 400-mesh copper grid at room temperature. To determine the mean particle size of rGO/Pd@PPy NPs and find integrated composition palladium on the support, approximately 300 particles were counted for the calculation. Panalitic Imperial diffractometer equipped with a high-resolution goniometer (with Cu Kα radiation (λ = 1.5418 Å), tube voltage of 45 kV and a tube current of 40 mA) was employed for X-ray diffraction (XRD) analysis. XRD patterns were recorded in the 2θ range of 10–90°. XPS analyses of rGO/Pd@PPy NPs were performed with a thermal scientific spectrometer composed of Kα MgS line and X-ray source (10 mA, 253.6 eV). All the XPS analyses, 284.6 eV of C 1s was taken as reference, and Gaussian function was used for the peaks. Raman microprobe (Renishaw Instruments) with 514 nm laser excitation was used to obtain a Raman spectrum. The experimental set up for the electrochemical studies was consist of three electrodes of a GCE decorated with rGO/Pd@PPy NPs, Ag/AgCl as a reference electrode and platinum as the counter electrode. A phosphate buffer solution (0.1 M, pH 3.0) was used in the electrolytic system at room temperatures. Prior to electrochemical measurement, all electrolytes were deoxygenated with nitrogen bubbles for 1-2 minutes and kept at an inert condition during the experiments. electrochemical impedance spectroscopy (EIS) analysis with conventional three electrodes technique was performed in a Gamry (Reference 3000) potentiostat/galvanostat with the frequency range of 1–10^5^ Hz in the electrolyte of 1.0 mM [Fe(CN)6]^3−/4−^ which contains 0.1 M KCl as supporting electrolyte. A platinum wire used as a counter electrode and Ag/AgCl (3 M KCl) used as a reference electrode under atmospheric conditions. The exposed surface area of the working electrode was 7.065 mm^2^. DPV experiments were conducted in a phosphate buffer solution at pH 3.0 and the electrical potential range was between −0.20 V and +0.80 V. For the detection of response values, the concentration of target molecules was varied, while the concentrations of the other two molecules were kept constant.

## Results and Discussion

### The characterization of rGO/Pd@PPy NPs

**Figure 2:**
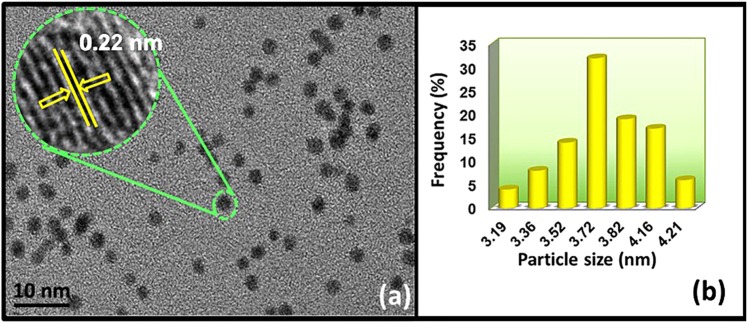
(**a**) TEM and HRTEM image, (**b**) particle size histogram of rGO/Pd@PPy NPs.

Pd particle distribution on the PPy/rGO and the average particle size of rGO/Pd@PPy NPs were investigated by transmission electron microscopy (TEM) and high-resolution electron microscopy (HRTEM). The lattices within the area of green circle in Fig. 2(a) were perfectly parallel, and the d-distances of the adjacent fringes were measured. According to HRTEM micrographs, rGO/Pd@PPy nanoparticles were in spherical-shape and abundant in the form of black spots. There is no clumping between the particles, and Pd NPs were equally distributed over rGO/PPy support. Figure 2(b) shows that the atomic lattice fringes obtained from TEM analysis of rGO/Pd@PPy NPs, and the plane pitch Pd (111) was found to be 0.22 nm corresponding to the nominal Pd (111) value. As a result, it was found that the prepared nanoparticles prepared in a relatively narrow range on rGO/PPy support and particle size were 3.70 ± 0.53 nm.

**Figure 3:**
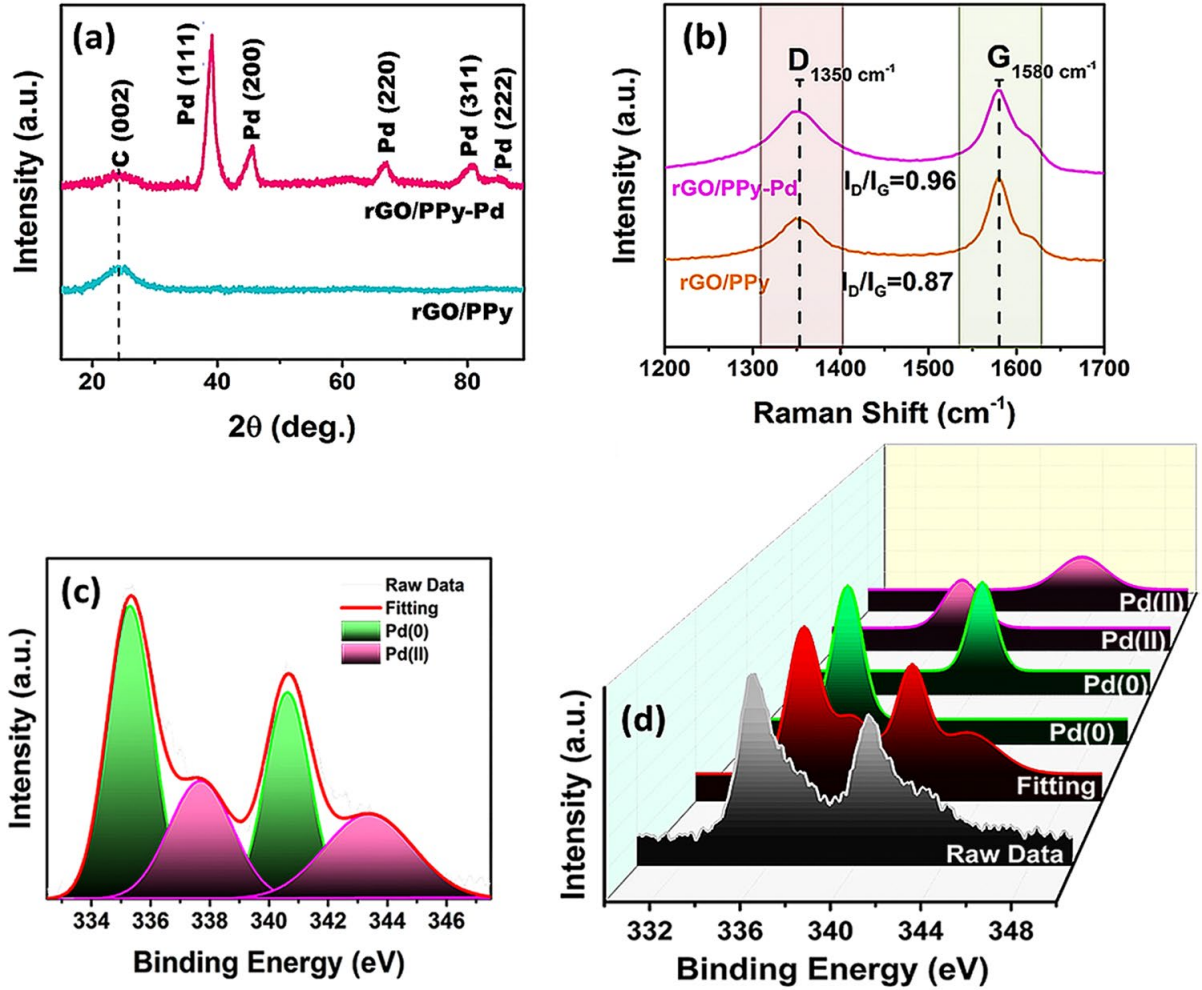
(**a**) XRD spectra NP and (**b**) Raman spectra of rGO/Pd@PPy and rGO/PPy. **(c**) XPS spectrum of Pd (0) and Pd (II). (**d**) The related 3D image of the XPS spectrum.

XRD diffractogram of rGO/Pd@PPy NPs and PPy/rGO are seen in Fig. 3(a). The diffraction lines at about 2θ = 39.82, 46.35, 67.72, 81.49 and 87.26 originate from planes (111), (200), (220), (311) and (222) corresponding to the Pd NPs of the face-centered cubic (fcc) crystal lattice. The diffraction peak at about 2θ = 24.50 corresponds to the mixture of reduced graphene oxide and PPy. A weak rGO/PPy diffraction peak was observed in rGO/Pd@PPy diffractogram, as a result of that the strong diffraction peak of Pd has overshadowed the weak carbon peaks. Scherrer equation and TEM patterns were used to calculate the average size Pd NPs on the support material. The diffraction peak (220) for palladium present on the prepared nanoparticles of PPy/rGO was measured to detect the lattice parameter value and the mean crystallite size of the palladium nanoparticles. The lattice parameter was calculated as 3.910 Å in agreement with the reported value of 3.923 Å for pure Pd^32,33^. The mean crystallite size of rGO/Pd@PPy nanoparticle was calculated to be 3.70 ± 0.53 nm using the Scherer formula.

Raman spectroscopy is an operative technique for investigating structures containing carbon. The Raman spectrum of rGO/PPy and rGO/Pd@PPy is shown in Fig. 3(b). The prepared nanocomposite revealed a typical G band and D bands at 1580 cm^−1^ and 1350 cm^−1^, respectively. It is associated with the existence of sp^3^ defects from the rGO, also the tangential vibration of the sp^2^ C atoms in the hexagonal plane, respectively. I_D_/I_G_ ratio for rGO/PPy and rGO/Pd@PPy was found to be 0.87 and 0.96, respectively. This indicates that rGO is functional with PPy-Pd. In addition, the increase in I_D_/I_G_ ratio proves that rGO/Pd@PPy hybrid was successfully synthesized^32,34^.

XPS technique is used to evaluate the surface metal composition and chemical oxidation status of the rGO/Pd@PPy NPs. The core levels (as seen in Fig. 3(c,d)) for Pd 3d_5/2_ at 335.8 eV indicate the presence of a metallic form of palladium in the rGO/Pd@PPy NPs. Additionally, the binding energy values obtained from our experimental results are compatible with the findings in the literature^35,36^. The peak seen at 337.9 eV indicates Pd(II), this might be as a result of the oxidations on the surface of rGO/Pd@PPy NPs, during the nanoparticles preparation process (Fig. 3(c,d)). XPS analysis revealed that metallic Pd° is dominantly existing, rather than Pd (II).

### The effect of scan speed

**Figure 4:**
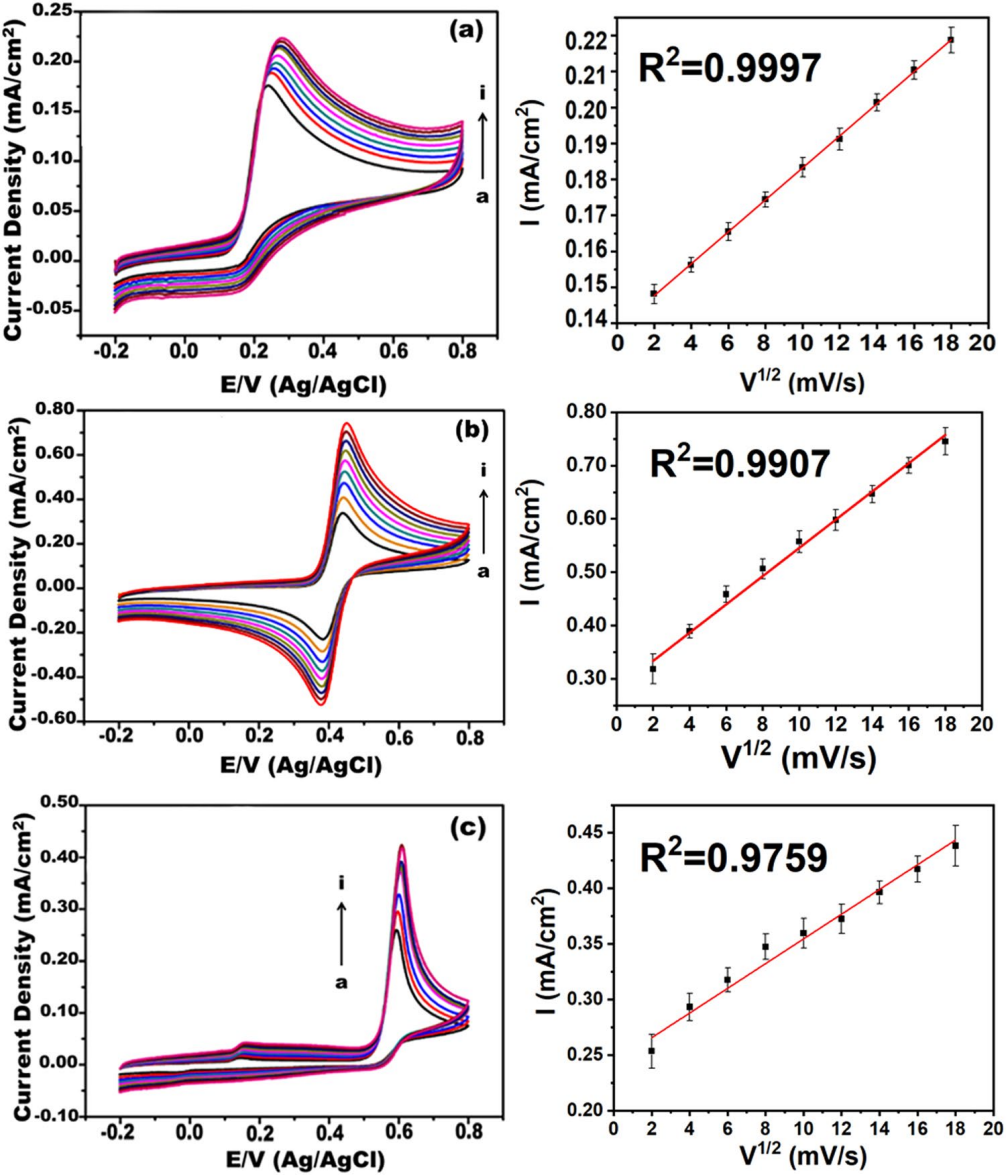
Cyclic voltammograms of 2.5 × 10^−3^ M of (**a**) AA, (**b**) DA and (**c**) UA at various scan rates (10 to 90 mVs^−1^) at rGO/Pd@PPy/GCE and corresponding Ip Vs ν^1/2^ plots.

The electrochemical response of the working electrode contains rGO/Pd@PPy NPs was investigated at different scan speeds using CV. The oxidation current peaks are given in Fig. 4. According to results, the increasing speed provides an increase in the intensity of the oxidation peaks, but the completion of the catalytic reaction is restricted due to the limited time. Although the oxidation peak values are increased, there are some limitations in the reaction kinetics between the target molecules and chemicals in the redox regions on the modified electrodes. The plots created using the obtained anodic peak currents versus (Ip) the square root of the scanning speed (V^1/2^, 10-90 mV s^−1^) values gives a better linear graphics compared to Ip versus V. The chemical reaction occurred among these molecules on the electrodes is compatible with the diffusion-controlled model.

### The effect of pH

**Figure 5:**
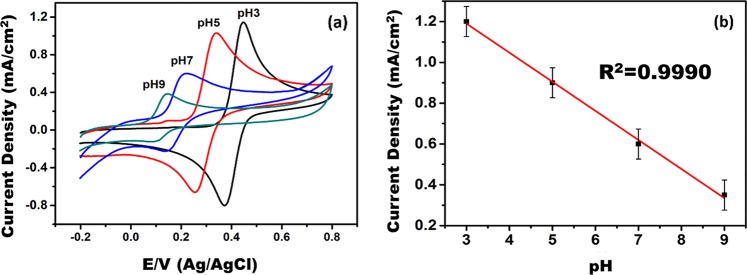
Cyclic voltammograms of 5 × 10^−3 ^M of DA at different pH values (3.0, 5.0, 7.0, and 9.0).

In order to investigate the effects of pH on electrochemical responses of 5 × 10^−3^ M of AA, DA, and UA, phosphate buffers at pH values of 3.0, 5.0, 7.0, and 9.0 were prepared. The results of the experiment given in Fig. 5 showed that there was a significant decrease in the cationic and anionic peak potentials of DA with increasing pH values at the modified electrode. This indicates that the presence of transferred protons in the generated DA redox reaction. A linear plot of DA versus pH in a range of 3.0‒9.0 with a 60 mV pH^−1^ of the slope was obtained from the results of the experiments as given in Fig. 5(b). The obtained slop value corresponds to the theoretical value of 59 mV pH^−1^ (25 °C). Additionally, a presence of equal numbers protons and electrons is seen because of the oxidations reaction occurred between DA and rGO/Pd@PPy NPs. In the pH effect experiments, the maximum responses in anodic current for AA, DA, and UA were obtained at pH 3.0. In the light of this result, to ensure taking simultaneous detection of AA, DA, and UA, the pH of 0.1 M phosphate buffer was adjusted to 3.0.

### The effect of different compositions on electrode responses

**Figure 6:**
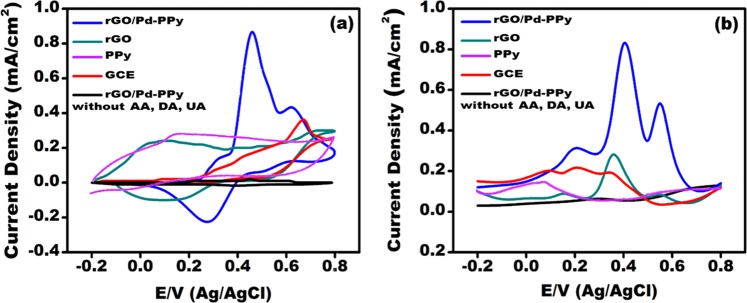
(**a**) CV responses of rGO/Pd@PPy modified electrode (3 × 10^−3^ M each; scanning speed of 50 mV s^−1^) (**b**) DPV curves (3 × 10^−3^ M each, pH 3.0, from −0.20 to 0.80 V.

The CV and DPV methods were used in the electrocatalytic studies of rGO/Pd@PPy NPs. The responses to AA, DA, and UA was measured to demonstrate the performance and efficiency of the fabricated electrode. Figure 6 shows the comparative CV and DPV curves of each component rGO/Pd@PPy NPs, rGO, GCE, PPy and blank rGO/Pd@PPy NPs (without AA, DA, and UA). The experiments performed using 3 × 10^−3^ M of AA, DA, and UA. It was found that the presence of AA and UA was not affected by the detection of DA at pH 3.0. The best-separated oxidation peaks were observed at 0.31 V, 0.48 V, and 0.61 V for AA, DA, and UA, respectively. The highest current density was observed at rGO/Pd@PPy modified electrode and had peak current values of 180 μA, 820 μA, and 410 μA for AA, DA, and UA, respectively (Fig. 6(a)). The results revealed that the electrodes modified with rGO/Pd@PPy NPs exhibited superior resolution, high sensitivity, and electrochemical potential in the electrochemical detection of AA, DA, and UA.

### Electrochemical impedance characterization of electrodes

**Figure 7:**
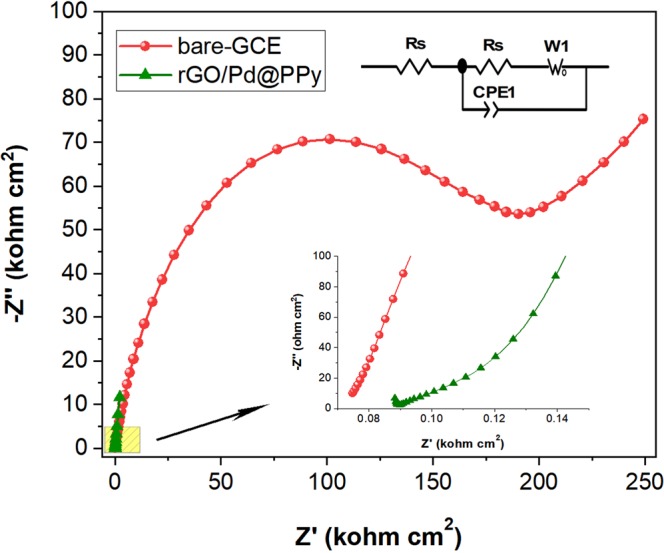
Nyquist plots of bare-GCE and rGO/Pd@PPy modified electrodes.

Electrochemical impedance spectroscopy analysis has been performed to explore the electron transfer kinetics of bare GCE and rGO/Pd@PPy modified electrode. The Nyquist plots of bare GCE and rGO/Pd@PPy were represented in Fig. 7. The electron transfer resistance (R_CT_) of the electrodes has been calculated by fitting data using Gamry Echem Analyst software. The R_CT_ values were found to be 640700 Ω cm^2^ and 98 Ω cm^2^ at bare-GCE and rGO/Pd@PPy modified electrode. The comparison of each component in the developed electrode was shown in Fig. S3. The RCT values of rGO and PPy were determined as 357 Ω cm^2^ and 258 Ω cm^2^. This significant drop in Rct value indicates that it has better electron conductivity and electrochemical properties compared to the bare-GCE electrode, and the results are in good agreement with the CV measurements.

### The use of rGO/Pd@PPy NPs modified electrode for individual and simultaneous detection of AA, DA, and UA

**Figure 8:**
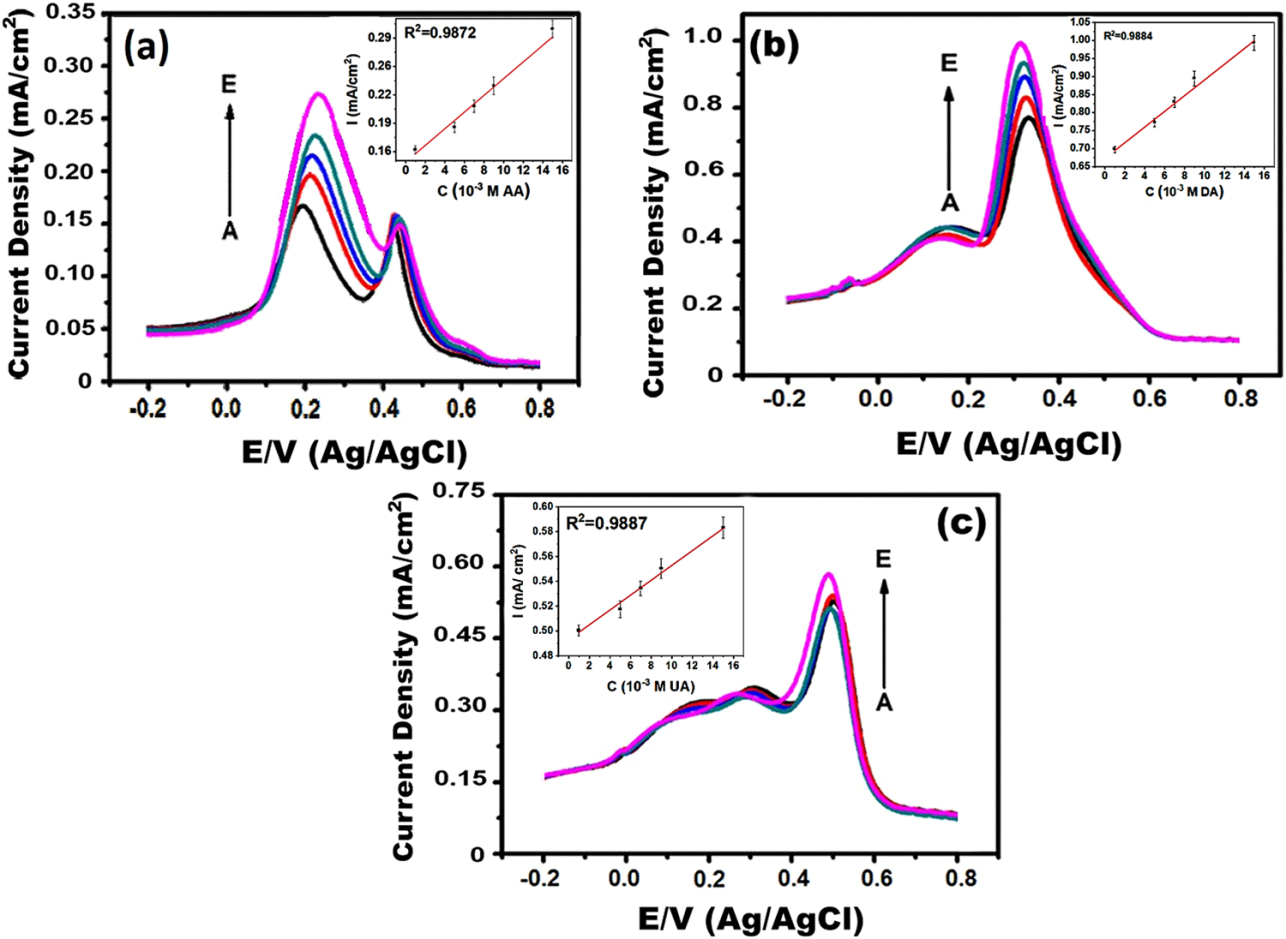
(**a**) DPV results in the presence of 1 × 10^−3^ M UA and 1 × 10^−3^ M DA at different concentrations of AA (**b**) DPV results in the presence of 1 × 10^−3^ M AA and UA at different concentrations of DA (**c**) DPV results in the presence of 1 × 10^−5^ M DA and 1 × 10^−4^ M AA at different concentrations of UA. (A: 0.5 × 10^−3^ M, B: 1 × 10^−3^ M, C: 1.5 × 10^−3^ M, D: 2 × 10^−3^ M, and E: 3 × 10^−3^ M).

The studies of AA, DA, and UA are shows that these molecules always present together in human body fluids. For this reason, rGO/Pd@PPy NPs used for the simultaneous and individual detection of AA, DA, and UA. DPV process was conducted to investigate the performance of electrodes modified with rGO/Pd@PPy NPs. The DPV curves obtained at different concentrations for AA, DA, and UA were given in Fig. 8(a–c). The experimental study was based on the fluctuating the concentration of target biomolecule, while the concentrations of other biomolecules were unchanged. The specific electrochemical oxidation currents were increased with the addition of the target biomolecule. However, the oxidation peak currents of other biomolecules were not increased significantly, and not interfere with each other. This indicated that the presence of well-defined oxidative peaks for each molecule (0.20, 0.35 and 0.49 V according to DPV results). The linear detection values of AA, DA, and UA, detection limit, etc. were obtained individually at rGO/Pd@PPy modified electrode are listed in Table S1. The modified electrode is remained stable for weeks and has very good sensitivity for AA, DA, and UA, respectively.

**Figure 9:**
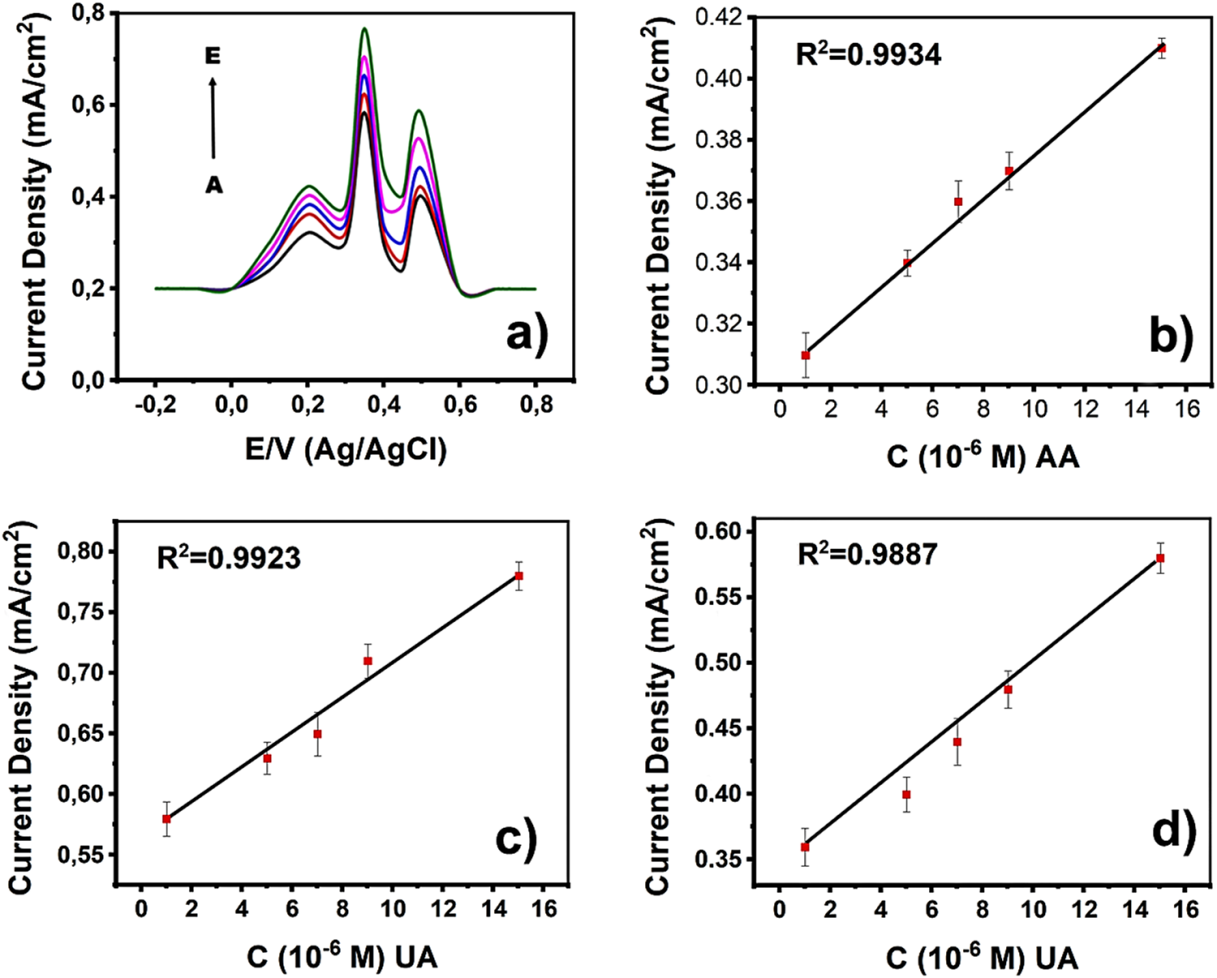
(**a**) DPV profiles in rGO/Pd@PPy-GCE at different concentrations of AA, DA, and UA (A: 1 × 10^−3^, B: 1.5 × 10^−3^, C: 2 × 10^−3^, D: 2.5 × 10^−3^ M, and E: 3 × 10^−3^ M), at pH 3.0. Associated calibration plots of (**b**) AA, (**c**) DA, and (**d**) UA.

The simultaneous detection of AA, DA, and UA was achieved at pH 3.0 using PBS, the potential range was from −0.20 to 0.80 V. The simultaneous detection tests were carried out by changing the concentrations of all the three analytes. DPV results and related calibration graphs for the determination of AA, DA, and UA were shown in Fig. 9. The minimum detection limit and linear detection range rGO/Pd@PPy for each analyte were calculated in the existence of the other two analytes, as shown in Table S1. The linear range of rGO/Pd@PPy is up to 12000, 38–1647 and 1.4–219 µM for AA, DA, and UA, respectively. The detection limits were found to be 0.049; 0.056; 0.047 µM for AA, DA, and UA, respectively. A comparison of the characteristics of the electrochemical sensors utilized for the determination of AA, DA, and UA simultaneously was given in Table S1. Our findings support that rGO/Pd@PPy nano-enhanced sensor, developed in this study, has very good efficiency and good sensitivity and for the simultaneous detection of UA, DA, and AA.

### Effect of repetitive voltammetric cycles

There are many studies on the voltammetric determination of dopamine using carbon-containing materials due to suitable potential ranges. However, carbon electrodes can be easily contaminated. Dopamine electrooxidation is known to cause contamination on the electrode^37^. We also studied the contamination effect that affects the susceptibility of AA, DA, and UA. For this aim, rGO/Pd@PPy NPs modified and bare GCE electrodes tested by recording sequential CV measurement (0.1 V s^−1^ scanning speed at pH 3.0 and 7.0). Figure S1 shows typical CVs of modified rGO/Pd@PPy and bare GC electrodes for the repeated cycle at 1 × 10^−3^ M DA (0.1 M phosphate buffer, pH 3.0 and 7.0). A fouling effect can be seen on the graph at pH 7.0, however, this effect significantly reduced at pH 3.0. This may be a result of electrooxidation of DA at higher pH values, leading to the producing of poly-DOPA on the working electrode and decreasing of electrocatalytic activity and sensitivity of electrode with increasing scans numbers. Additionally, AA and UA oxidation effects for the detecting sensitivity of DA by using DPV and CV at the experimental conditions were studied. The repeating CVs for the electrooxidation of DA is given in Fig. S2A–C, separately and together, in the presence of 1 × 10^−3^ M of AA and UA. To obtain a percentage reduction of the available signals, the corresponding DPV profiles were also obtained under optimal conditions (Fig. S2D). It can be concluded that the sensitivity of DA is affected slightly with the increasing scan numbers and electro-oxidation of DA with increasing scan numbers have affected the sensitivity of AA and UA. All of these findings indicate that the prepared rGO/Pd@PPy NPs can be effectively used in various applications.

### Reproducibility studies of modified electrodes with rGO/Pd@PPy NPs

rGO/Pd@PPy NPs samples were stored at room conditions, and CV-DPV measurements were performed in every ten days (for 3 months) to assess the electrode stability. In order to get the responses of rGO/Pd@PPy NPs modified electrodes for AA, DA, and UA, a dilute serum sample were prepared in a phosphate buffer. Approximately, 200 ml serum solution was used in the recovery experiments. The content of AA, UA, and DA in the rGO/Pd@PPy/GCE serum sample was used to control the feasibility of electrochemical analysis. The prepared serum samples transferred into the electrolytic reaction medium, and the electrochemical experiment was initiated. The results showed that the relative standard deviation (R.S.D.) was found to be anodic oxidation peak currents of 4.2%, 3.3%, and 2.7%, for AA, DA, and UA, respectively. These findings demonstrate the excellence of reproducibility and good stability rGO/Pd@PPy NPs. This indicates that the fabricated sensor can be utilized in real serum samples. The results are shown in Table 1. Recoveries obtained at different concentration levels, in the range of 94.4–101%, shows no significant interaction between the modified electrodes and the serum samples. These results indicate that the fabricated rGO/Pd@PPy NPs are effective material in the detection application of AA, DA, and UA using serum samples.

**Table 1:** The experimental results obtained from the detection studies using 200 mL serum sample. (The experimental conditions of DPV measurements: 180 sec, scan range −0.20 to +0.80 V, pH 3.0).

Serum sample	Found (10^−6^ M)			Spiked (10^−6^ M)			Recovery (10^−6^ M)			% Recovery		
	AA	DA	UA	AA	DA	UA	AA	DA	UA	AA	DA	UA
I	n/a	n/a	33.0	100	10	10	95 ± 1.2	9.7 ± 0.9	41.9 ± 0.3	95	97	97.4
II	n/a	n/a	34.0	250	25	25	241 ± 0.4	23.6 ± 0.3	59.8 ± 0.2	96.4	94.4	101.4
III	n/a	n/a	33.0	500	50	50	485 ± 0.3	48.2 ± 0.3	82.9 ± 0.9	97	96.4	99.9

^*^n/a: not detected or below the detection limits.

Some features including linear calibration and the detection limits for AA, DA, and UA, are given in Table S1 and compared to previous studies in the literature. This research supports that rGO/Pd@PPy NP modified electrodes exhibited a high relative detection limit and a better linear response to DA when compared to the GCE.

## Conclusion

A facile method was developed to prepare the electrodes modified with rGO/Pd@PPy NPs. The modified electrode was utilized for the individual and simultaneous detection of AA, DA, and UA, which can be useful for the early diagnosis of neurodegenerative diseases. rGO/Pd@PPy/GCE electrode showed a significant peak current that demonstrated synergistic effects between rGO, Pd, and PPy. The prepared rGO/Pd@PPy NPs on the electrodes exhibited excellent electrocatalytic performance, effective electron transfer capability, and better sensitivity compared to the previous findings in the sensing of AA, DA, and UA. Under optimum conditions, the produced electrode has shown good selectivity, high stability, and a wide linear concentration range. (LOD 4.9 × 10^−8^, 5.6 × 10^−8^, 4.7 × 10^−8^ M and LOQ 1.6 × 10^−7^, 1.8 × 10^−7^ and 1.5 × 10^−7^ M). In addition, the developed electrochemical sensor can effectively sense AA, DA, and UA concentrations in real serum samples. This study illuminates future developments with the presence of other biomolecules in the separate and instantaneous measurement of AA, DA, and UA.

## Supplementary information

Supplementary information.

Dataset 1.

Dataset 2.

Dataset 3.
