# Supplementary material for: Palladium supported on polypyrrole/reduced graphene oxide nanoparticles for simultaneous biosensing application of ascorbic acid, dopamine, and uric acid
Source: Sci Rep. 2020 Feb 19;10:2946. doi: 10.1038/s41598-020-59935-y (PMC7031288; doi:10.1038/s41598-020-59935-y)
Supplement: Supplementary file 1 — Supplementary information. [file 41598_2020_59935_MOESM1_ESM.docx]

**Supporting information**

**Palladium supported on polypyrrole/reduced graphene oxide nanoparticles for simultaneous biosensing application of ascorbic acid, dopamine, and uric acid**

**Buse Demirkan^a^, Sait Bozkurt^a^, Kemal Cellat^a^, Kubilay Arıkan^a^, Mustafa Yılmaz^a^, Aysun Şavk^a^, Mehmet Harbi Çalımlı^a,b^, Mehmet Salih Nas^a,b^, Mehmet Nuri Atalar ^b^, Mehmet Hakkı Alma^b^, Fatih Sen*^a^**

^a^Sen Research Group, Department of Biochemistry, Faculty of Arts and Science, Dumlupinar University, Evliya Çelebi Campus, 43100 Kütahya, Turkey.

^b^Department of Environmental Engineering, Faculty of Engineering, University of Igdir, Igdir, Turkey.

^*^Corresponding author: fatih.sen@dpu.edu.tr

Tel:90 274 265 20 31 -37 02 Fax:90 274 265 20 56

**Materials**

The chemicals including graphite powder, pyrrole monomer, cetyltrimethylammonium bromide (CTAB) and ammonium persulfate (APS), K_2_PdCl_4_, dimethyl sulfoxide (DMSO), sodium nitrite (NaNO_2_), potassium ferrocyanide (K_4_Fe(CN)_6_), AA, DA, and UA were obtained from Sigma-Aldrich, ethanol was provided from Merck with their purity in analytical grade. Pyrrole monomer was purified by distillation pirior to experiments. Other chemical reagents were analytical grade and used as received without any further purification. The pure water was obtained by a commercially available instrumentation (Millipore Milli-Q Ultrapure Ion Organic-Free Water System). All the glassware used in the experiments were cleaned with acetone and distilled water. Phosphate buffer solution (PBS, pH 3.0, 0.1 M (mol.L^-1^)) was prepared by potassium dihydrogen phosphate (KH_2_PO_4_) and sodium hydroxide (NaOH).

**Apparatus**

At room temperature, a three-electrode cell was utilized during electrochemical tests using a potentiostat/galvanostat Gamry Interface 3000 P/G system (USA). The working electrode was a GC disc. A saturated Ag/AgCl reference electrode, and a platinum wire used as auxiliary electrode.

**Electrochemical studies**

AA, DA, UA solutions were introduced into an electrochemical cell having 0.1 M phosphate buffer, after that three electrodes were placed. Experiments were conducted in buffer medium at pH 3.0. Potential range was from -0.20 to +0.80 V, current evaluation was performed by DPV. Scan rate of 50 mV/s, pulse with of 0.1 s and pulse amplitude of 2 mV were employed during DPV measurements. The amounts of UA, AA, and DA were determined simultaneously by evaluating the oxidation peak currents versus the amount of analytes.

**The preparation of Graphene Oxide (GO) and Reduced Graphene Oxide (rGO)**

The synthesis of graphite oxide was accomplished by modified Hummer's method using graphite particles. Typically, 5.0 g of graphite (<45 mm, Sigma- Aldrich) and 180 mL of concentrated sulfuric acid was mixed for one hour in a ventilated compartment. After that, nitric acid (60 mL) and potassium permanganate (25 g) was introduced slowly, while the mixture was cooling with ice. The resulting semiliquid solution was mixed in a ventilated compartment for 120 min. 600 mL water added mixed for two hours, 30 mL hydrogen peroxide (30%) was poured, bright yellow coloured-mixture formed. The resultant centrifuged and washed with water (1000 mL), HCl (5 mL, 37%) and hydrogen peroxide (3 mL, 30%). The application above-mentioned was carried out for three times. In order to get neutral pH value, the mixture was washed with certain amount of water (500 mL). The resulting dark-yellow particles were dried at 40 ^o^C under vacuum for 48 hours. Due to the decomposition of graphene oxide over 60-80  ^o^C , it should be dried at lower temperatures. rGO was prepared as follows: 100 mg GO was loaded in a 250 mL water then homogeneous dispersion was sonicated for 1 h. Hydrazine hydrate (1.00 mL, 32.1 mmol) was added into the solution (1 mg GO) and refluxed at 100-120 ^o^C. The rGO was obtained as a black solid material. It was filtered then, washed with water and ethanol, and dried under vacuum at 80 ^o^C.

**Preparation of electrodes**

Glassy carbon (GC) electrodes were used as substrates for supported catalysts. GC was 3 mm in diameter, and polished using 0.05 mm alumina to a mirror-finish, prior to experiments. Preparation of electrodes was done as following describtion: 3 mg of rGO/Pd@PPy was added into 0.5 mL of (0.05% wt.) Nafion solution, and the mixture was placed in an ultrasonic bath for 1 h to obtain a uniform dispersion. 8 µL of this mixture was dropped by a microsyringe onto the top surface of the GC. The catalyst-modified GC electrode was employed as the working electrode in the experiments.


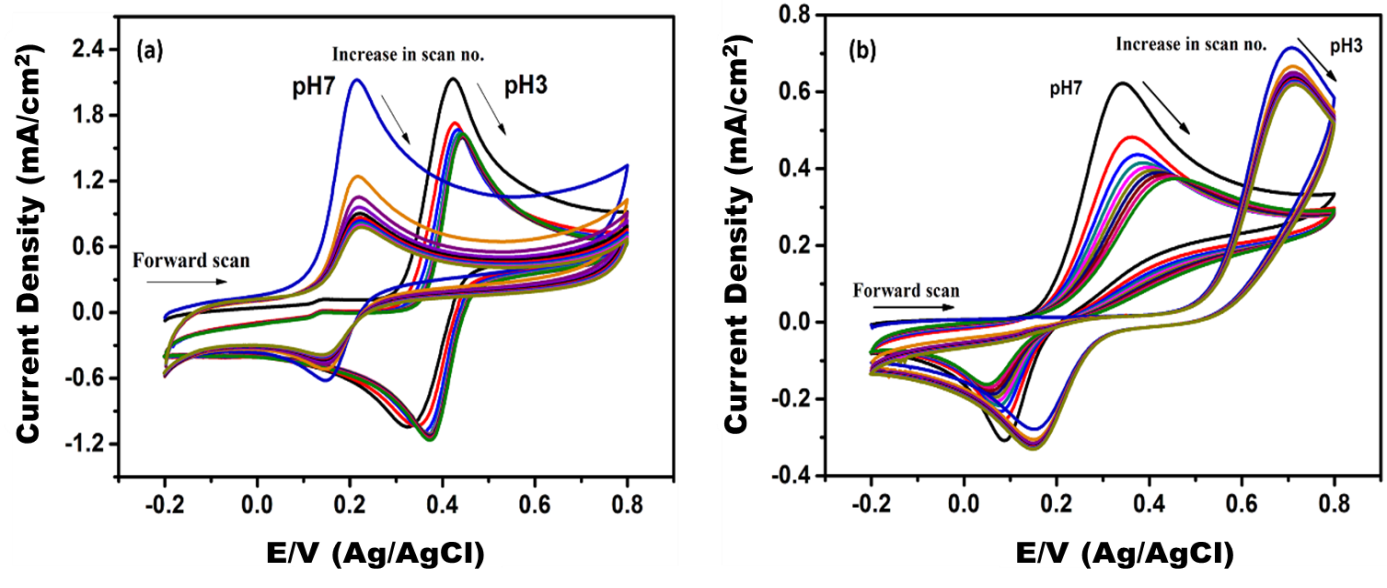


**Figure S1.** Repetitive cycling for the electro-oxidation of 5×10-3M DA in 0.1 M phosphate buffer at pH 3.0 & 7.0 (a) rGO/Pd@PPy/GCE (b) Bare GCE.

**
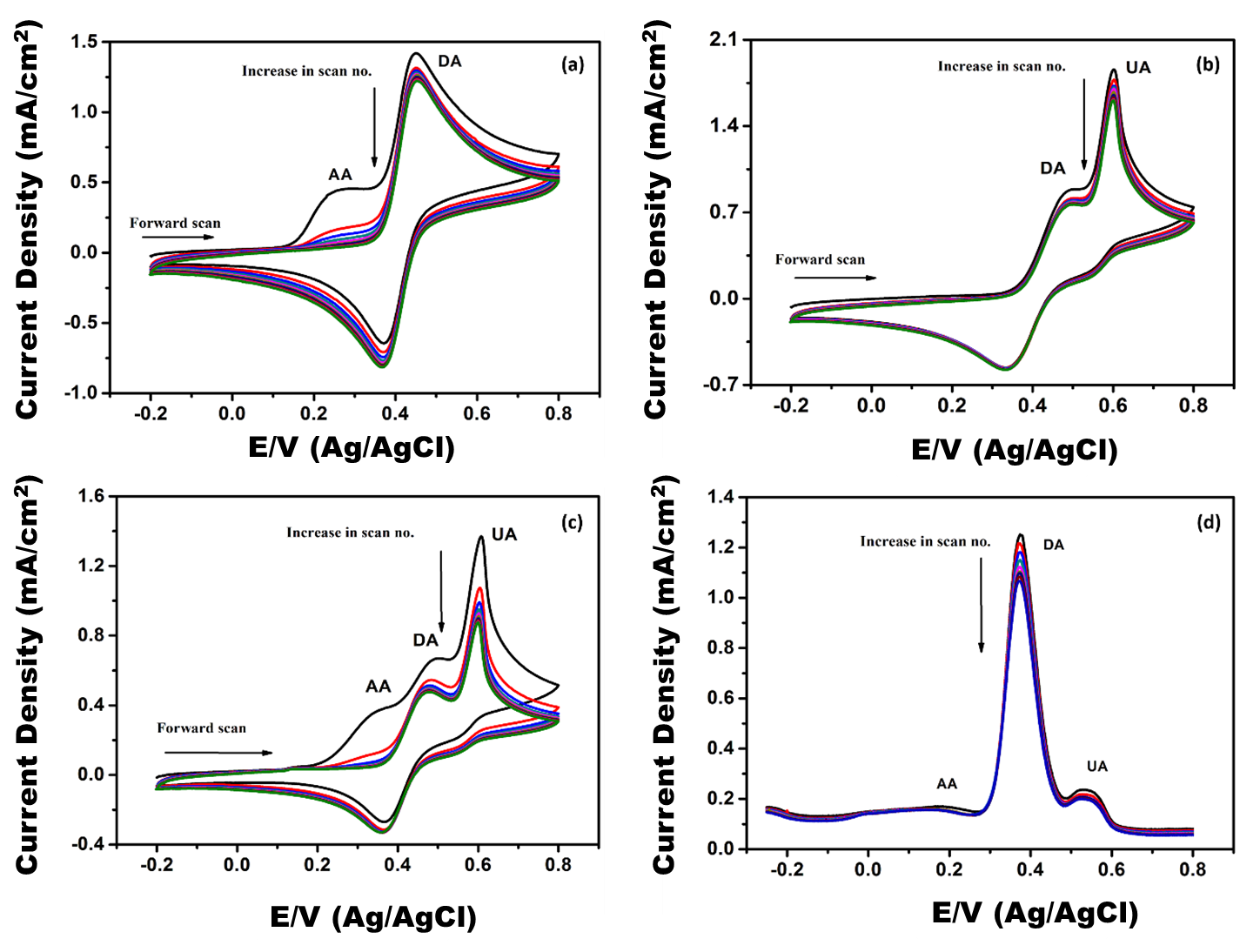
**

**Figure S2.** Repetitive cycling for the simultaneous electro-oxidation of 5× 10^-3^ M of (a) AA, (b) DA, (c) UA in phosphate buffer at pH 3.0, and (d) corresponding DPV profile.


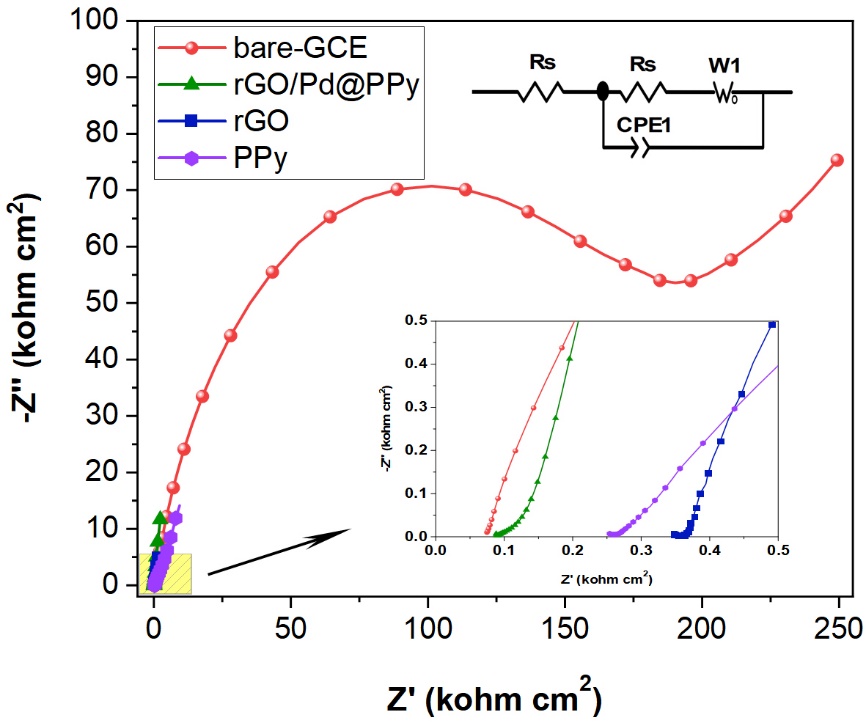


**Figure S3.** Comparison of Nyquist plots of each components.

**Table S1**: Comparison of recently reported electrochemical sensors for individual and simultaneous determination of AA, DA and UA.

| **Electrode** | **Linear range (10-6M)** | | | **Detection Limit (10-6M)** | | | **References** |
| --- | --- | --- | --- | --- | --- | --- | --- |
|  | **AA** | **DA** | **UA** | **AA** | **DA** | **UA** |  |
| Nano-Cu- PSAIII/GCE | 0.30-730 | 0.02-65 | 0.25-107 | 0.15 | 0.01 | 0.10 | [1] |
| pristine graphene | 9.00 –2314 | 5.00 – 710 | 6.00 –1330 | 6.45 | 6.45 | 4.82 | [2] |
| hierarchical nanoporous PtTi alloy | 0.2 - 1 | 0.004 - 0.5 | 0.1 - 1 | 24.2 | 3.2 | 5.3 | [3] |
| Fe_3_O_4_-SnO_2_-Gr | 0.1 -23 | 0.02 - 2.8 | 0.015 - 2.40 | 62.0 | 7.1 | 5.0 | [4] |
| Graphene ink | 50 –1000 | 3 – 140 | 0.5 - 150 | 17.8 | 1.44 | 0.29 | [5] |
| rGO incorporated L-lysine | 100 –1200 | 2 – 60 | 20 -200 | 2000 | 100 | 150 | [6] |
| Zn-NiAl LDH/rGO superlattice | 0.5 – 11 | 0.001 – 1 | 0.0011 – 0.95 | 13.5 | 0.1 | 0.9 | [7] |
| N_2_/Ar/GS/GNR | 0.1 –1400 | 0.01 –400 | 0.02–350 | 0.0053 | 0.0025 | 0.0057 | [8] |
| ZnNi@f-MWCNT | 200 -1200 | 250 -1700 | 200 -900 | 0.5110 | 0.0655 | 0.0882 | [9] |
| PPy hydrogel/GCE | 2.5 –1500 | 0.08–250 | 0.25–400 | 1.283 | 0.044 | 0.046 | [10] |
| rGO/Pd@PPy -GCE | **Up to 12000** | **38-1647** | **1.4-219** | **0.049** | **0.056** | **0.047** | **This study** |

**REFERENCES**

[1] L. Zhang, W.-J. Yuan, and B.-Q. Hou, ‘Nano-Cu/PSA III modified glassy carbon electrode for simultaneous determination of ascorbic acid, dopamine and uric acid’, *J. Electroanal. Chem.*, vol. 689, pp. 135–141, Jan. 2013.

[2] S. Qi, B. Zhao, H. Tang, and X. Jiang, ‘Determination of ascorbic acid, dopamine, and uric acid by a novel electrochemical sensor based on pristine graphene’, *Electrochim. Acta*, vol. 161, pp. 395–402, Apr. 2015.

[3] D. Zhao, G. Yu, K. Tian, and C. Xu, ‘A highly sensitive and stable electrochemical sensor for simultaneous detection towards ascorbic acid, dopamine, and uric acid based on the hierarchical nanoporous PtTi alloy’, *Biosens. Bioelectron.*, vol. 82, pp. 119–126, Aug. 2016.

[4] H. Bagheri, N. Pajooheshpour, B. Jamali, S. Amidi, A. Hajian, and H. Khoshsafar, ‘A novel electrochemical platform for sensitive and simultaneous determination of dopamine, uric acid and ascorbic acid based on Fe3O4SnO2Gr ternary nanocomposite’, *Microchem. J.*, vol. 131, pp. 120–129, Mar. 2017.

[5] L. Fu *et al.*, ‘Defects regulating of graphene ink for electrochemical determination of ascorbic acid, dopamine and uric acid’, *Talanta*, vol. 180, pp. 248–253, Apr. 2018.

[6] D. Zhang, L. Li, W. Ma, X. Chen, and Y. Zhang, ‘Electrodeposited reduced graphene oxide incorporating polymerization of l -lysine on electrode surface and its application in simultaneous electrochemical determination of ascorbic acid, dopamine and uric acid’, *Mater. Sci. Eng. C*, vol. 70, no. Pt 1, pp. 241–249, Jan. 2017.

[7] M. Asif *et al.*, ‘Superlattice stacking by hybridizing layered double hydroxide nanosheets with layers of reduced graphene oxide for electrochemical simultaneous determination of dopamine, uric acid and ascorbic acid’, *Microchim. Acta*, vol. 186, no. 2, p. 61, Feb. 2019.

[8] L. Jothi, S. Neogi, S. kumar Jaganathan, and G. Nageswaran, ‘Simultaneous determination of ascorbic acid, dopamine and uric acid by a novel electrochemical sensor based on N 2 /Ar RF plasma assisted graphene nanosheets/graphene nanoribbons’, *Biosens. Bioelectron.*, vol. 105, pp. 236–242, May 2018.

[9] A. Savk *et al.*, ‘Multiwalled carbon nanotube-based nanosensor for ultrasensitive detection of uric acid, dopamine, and ascorbic acid’, *Mater. Sci. Eng. C*, vol. 99, pp. 248–254, Jun. 2019.

[10] M. Wang, M. Cui, W. Liu, and X. Liu, ‘Highly dispersed conductive polypyrrole hydrogels as sensitive sensor for simultaneous determination of ascorbic acid, dopamine and uric acid’, *J. Electroanal. Chem.*, vol. 832, pp. 174–181, Jan. 2019.
